# TRAIL Modulates the Immune System and Protects against the Development of Diabetes

**DOI:** 10.1155/2015/680749

**Published:** 2015-02-18

**Authors:** Fleur Bossi, Stella Bernardi, Giorgio Zauli, Paola Secchiero, Bruno Fabris

**Affiliations:** ^1^Department of Medical, Surgical, and Health Sciences, University of Trieste, Cattinara University Hospital, Strada di Fiume 447, 34100 Trieste, Italy; ^2^Institute for Maternal and Child Health, IRCCS Burlo Garofolo, Via dell'Istria 65, 34100 Trieste, Italy; ^3^Department of Morphology, Surgery, and Experimental Medicine, LTTA Centre, University of Ferrara, Via Fossato di Mortara 66, 44100 Ferrara, Italy

## Abstract

TRAIL or tumor necrosis factor (TNF) related apoptosis-inducing ligand is a member of the TNF superfamily of proteins, whose best characterized function is the induction of apoptosis in tumor, infected, or transformed cells through activation of specific receptors. In nontransformed cells, however, the actions of TRAIL are less well characterized. Recent studies suggest that TRAIL may be implicated in the development and progression of diabetes. Here we review TRAIL biological actions, its effects on the immune system, and how and to what extent it has been shown to protect against diabetes.

## 1. Introduction

TRAIL or tumor necrosis factor (TNF) related apoptosis-inducing ligand is a member of the TNF superfamily of proteins, whose best characterized function is the induction of apoptosis in tumor, infected, or transformed cells through activation of specific receptors. There is accumulating evidence showing that TRAIL-induced apoptosis is not limited to transformed cells but may be also induced in primary cells, such as immune cells. TRAIL seems in fact involved in the regulation of various physiological and pathological processes of the innate and adaptive immune system.

Diabetes mellitus (DM) is a heterogeneous clinical syndrome featured by high levels of glucose. Whether it is type 1 DM (T1DM) or type 2 DM (T2DM) the immune system is involved in the development and progression of both. Interestingly, animal studies suggest that TRAIL might protect against diabetes development and progression. The multiple mechanisms underlying TRAIL protective effects against DM may involve not only TRAIL proapoptotic actions on autoreactive T cells but also the promotion of T regulatory cells, as well as newly characterized antiadipogenic and anti-inflammatory actions. Here we review TRAIL biological functions, its effects on the immune system, and how and to what extent it has been shown to protect against diabetes.

## 2. Overview on TRAIL Biology

### 2.1. TRAIL Biology

**Figure 1:**
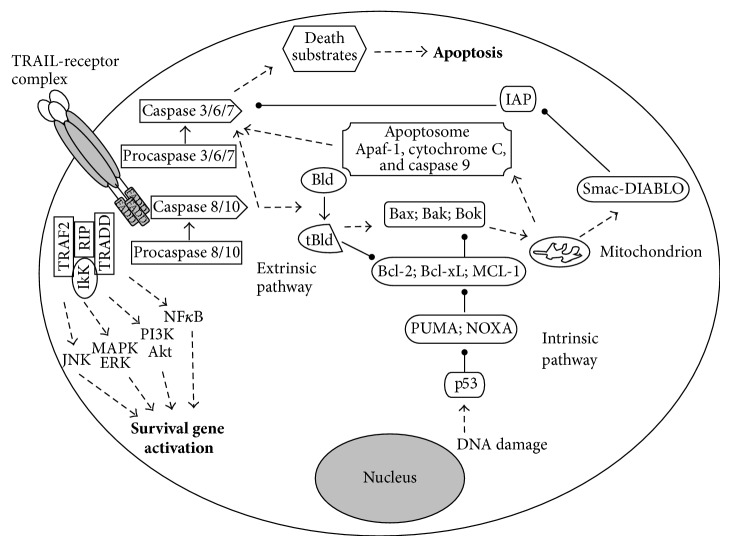
TRAIL-receptor mediated signalling pathways. By binding its receptor TRAIL initiates cell death (apoptosis) via either intrinsic (mitochondria) or extrinsic pathway and/or induces the activation of survival genes resulting in cell proliferation/migration and inhibition of apoptosis.

For a single-cell organism, life with mutations is apparently better than no life at all, but in multicellular organisms the health of the organism takes precedence over the life of an individual cell and, thus, when cells are damaged, altered, or they become unnecessary, they do not continue division but instead “commit suicide” by undergoing apoptosis. Apoptosis is a process leading to cell death, whereby unrequired cells can be eliminated in order to safeguard multicellular organism health. There are two ways of signalling leading to apoptosis. One is called “intrinsic pathway,” because it is triggered by an intracellular signal, such as DNA damage, while the other is called “extrinsic pathway,” because it is triggered by an extracellular signal, which usually derives from cytotoxic cells of the immune system, as in Figure 1 [1]. In particular, the extrinsic pathway is activated upon the binding of specific proapoptotic ligands, namely, FasL/CD95L and tumor necrosis factor-*α* (TNF-*α*), to their transmembrane receptors. This stimulates the trimerization of the transmembrane receptors and the formation of the death-inducing signalling complex (DISC), based on the recruitment of Fas associated death domain (FADD). Subsequently, FADD recruits both caspase-8 and caspase-10, which undergo autoactivation by proteolytic cleavage and which in turn activate caspase-3, caspase-6, and caspase-7, eventually executing the apoptotic program (Figure 1).

TRAIL is the third proapoptotic ligand that triggers the extrinsic pathway. The name TRAIL or TNF-related apoptosis-inducing ligand was chosen for the high homology of this protein to other TNF family members [2]. The percentage of identity with FasL/CD95L and TNF-*α* is in fact 28% and 23%, respectively. The gene encoding for TRAIL is located on chromosome 3 at position 3q26. TRAIL gene locus spans approximately 20 kb and it has five exonic segments and four introns. In humans, TRAIL is expressed as a type II transmembrane protein of 281 amino acids and it is composed of four parts: an extracellular TNF-like domain, an extracellular stalk, a transmembrane helix, and a small cytoplasmatic domain. After cleavage at the stalk domain, TRAIL is released as a soluble molecule with biological activity. It is at this stage that the cysteine residue at position 230 (Cys230) allows TRAIL to interact and assemble with other two molecules of TRAIL forming a trimeric ligand. These TRAIL homotrimers are proapoptotic agonists that bind to their specific death receptors on the surface of target cells and induce apoptosis (Figure 1). Recently, it has been shown that TRAIL can induce also necroptosis, which is a regulated and programmed form of necrosis that takes place after TRAIL binding to its specific death receptors and which can be useful to the body when apoptosis has been blocked [3, 4]. The human receptors for TRAIL, however, are not only death receptors (DR) but also decoy receptors (DcR). DR include TRAIL-R1 (DR4/TNFRSF10A) [5] and TRAIL-R2 (DR5/TNFRSF10B) [6–9], which are both type I transmembrane proteins containing an intracellular death domain (DD) that classically stimulates apoptosis upon TRAIL binding. As for DcR, they include TRAIL-R3 (TRID/DcR1/TNFRSF10C) [10], TRAIL-R4 (DcR2/TNFRSF10D) [11, 12], and osteoprotegerin (OPG) [13]. DcR1 and DcR2 are transmembrane receptors that differ from DR in that their cytoplasmatic domain lacks an intact DD, while OPG is a soluble receptor that lacks both transmembrane and cytoplasmatic residues.

### 2.2. TRAIL Biological Functions

The first and best characterized function of TRAIL is its ability to induce apoptosis in transformed cells, such as malignant cells. Studies on TRAIL-knockout mice have in fact demonstrated that mice without TRAIL are viable and fertile but more susceptible to tumor metastases, indicating that TRAIL regulates immune surveillance and host defence against tumor initiation and progression [14, 15]. In particular, TRAIL seems to mediate the ability of natural killer (NK) cells and cytotoxic T lymphocytes to block tumor growth and metastasis development [16, 17]. It should be noted that TRAIL-dependent killing by NK cells is believed to protect the organism also from infection [18].

Interestingly, one of the unique aspects of TRAIL, as compared to the other proapoptotic ligands [19, 20], is that TRAIL has the ability to induce apoptosis preferentially in transformed cells, such as tumor or infected cells, while it would spare most normal cells [21]. In particular Ashkenazi and colleagues demonstrated that the exposure of cynomolgus monkeys to recombinant human (rh) TRAIL at 0.1–10 mg/Kg/day over 7 days did not induce detectable toxicity, whereas, for comparison, TNF-*α* induced severe toxicity at much lower doses such as 0.003 mg/Kg/day [22]. The balance between TRAIL death and decoy receptors was initially considered a possible mechanism whereby cells could negatively regulate TRAIL-induced cytotoxicity, therefore explaining TRAIL selectivity. However, a correlation between this ratio and either protection or susceptibility to apoptosis has never been clearly demonstrated and other mechanisms have been put forward in order to explain the different sensitivity to TRAIL, such as the overexpression of c-FLIP, which could interfere with the activation of caspase-8 [23].

Another intriguing aspect of TRAIL is that it can apparently mediate also nonapoptotic signalling. A major contribution of our group to the biology of the TRAIL/TRAIL-R system was the discovery that when TRAIL R1/DR4 and TRAIL-R2/DR5 bind to TRAIL homotrimers they can stimulate not only proapoptotic pathways, but also prosurvival pathways, such as nuclear factor *κ*B (NF-*κ*B), ERK1/ERK2, and Akt [24, 25] (Figure 1). In particular the prosurvival signalling complex would still rely on DISC assembly, but instead of caspase activation, it would depend on the recruitment of TNF-receptor-associated death domain (TRADD), TNF-receptor-associated factor-2 (TRAF2), receptor interacting protein (RIP), and the inhibitor of *κ*B kinase (IKK*γ*). Once this complex has been assembled, it would then activate NF*κ*B, PI3K/Akt, and MAPKs, including ERK, as well as JNK and p38, leading to survival signals (Figure 1). It has been suggested that the ability of TRAIL to activate such opposed pathways depends on the incorporation of specific intracellular proteins into “lipid rafts.” Lipid rafts are platforms consisting of a dynamic pool of cholesterol and sphingolipids, which recruit signalling molecules including cell surface receptors. Several studies have reported that there is a redistribution of TRAIL receptors and DISC components from “non-rafts” into “lipid rafts” and it is current opinion that this is what could switch either cell apoptosis or survival upon TRAIL stimulation [26, 27]. Consistent with the concept that TRAIL triggers nonapoptotic signals in normal cells, we have recently demonstrated that systemic TRAIL delivery significantly reduced cardiac fibrosis and apoptosis in a mouse model of diabetic cardiomyopathy [28].

## 3. TRAIL and the Immune System

Besides the involvement in tumor surveillance and infection control, TRAIL seems to critically regulate also the immune system homeostasis. Animal studies have in fact shown that genetic loss of both FasL and TRAIL resulted in a condition that resembles to human autoimmune lymphoproliferative syndrome (ALPS), which is characterized by splenomegaly and lymphadenopathy, due to an accumulation of CD4^−^CD8^−^ “double negative” T cells [29]. The fact that in this model TRAIL deficiency affected the severity of lymphocyte accumulation indicates that TRAIL contributes to the control of peripheral lymphocyte apoptosis, perhaps in a secondary or cooperative manner with respect to FasL. In addition, the finding that TRAIL deficiency led to ALPS2 development, which is characterized by abnormal DC accumulation [23], suggests that TRAIL helps in getting rid of immature dendritic cells (DC), by killing them before lymph node entry.

Moreover, the observation that TRAIL-knockout mice had a severe defect in thymocyte apoptosis and were hypersensitive to both collagen-induced arthritis and streptozotocin-induced diabetes initially suggested that TRAIL could be critically involved in the maintenance of central tolerance by the negative selection of autoreactive thymocytes [30]. Nevertheless, further studies have challenged this hypothesis. Cretney and colleagues could in fact not reproduce the finding that negative selection was impaired in TRAIL-knockout mice [31], nor that the adaptor protein FADD was essential for thymic negative selection [32]. Eventually, though, Corazza and colleagues reconciled these conflicting results by suggesting that in the thymus TRAIL is a response modifier for mitochondrial apoptosis rather than a direct mechanism of thymic negative selection [33], therefore implying that TRAIL is involved, although only indirectly, in thymic apoptosis and thymic negative selection.

If central tolerance relies on thymic negative selection, peripheral tolerance is based on (i) anergy induction; (ii) the presence of regulatory CD4^+^CD25^+^ T cells (Treg); and (iii) the termination of T cell immune responses, which depends on the activation-induced cell death (AICD). It is in fact through AICD or by eliminating activated immune cells that the organism prevents any potential autoimmune damage. One of the first observations suggesting TRAIL involvement in mature lymphocyte poststimulation apoptosis was that TRAIL induced apoptosis in T cells after sensitization with IL-2 [34]. Subsequently, TRAIL was also found to play a role in AICD of human peripheral blood mononuclear cells [35], which is consistent with the concept that this molecule helps in getting rid of potentially dangerous immune cells. Apart from AICD, TRAIL seems to regulate peripheral tolerance also by promoting the proliferation of T regulatory (Treg) cells [36, 37]. These are cells that play an essential role in maintaining immune tolerance, and when they are absent, such as in individuals with IPEX (immune dysregulation, polyendocrinopathy, enteropathy, and X-linked syndrome), there is an enhanced susceptibility to autoimmune diseases and diabetes [38]. In addition to that, the third way whereby TRAIL could regulate peripheral tolerance is its ability to prevent primary T cell proliferation during the Ag-independent T cell activation [39].

Interestingly, it seems that TRAIL affects adaptive immune cells not only by inducing cell death, but also by inhibiting their activation and expansion [40]. On one hand, it has been shown that differentiating CD4^+^ Th1 cells [41] are selectively removed by TRAIL-mediated apoptosis [42], which explains why the frequency of CD4^+^ Th1 cells is greater in TRAIL-knockout mice with respect to their controls [36]. Likewise, also CD8^+^ Ag-activated T cells, generated in the absence of CD4^+^ T cells, are removed by TRAIL-mediated apoptosis [16]. On the other hand, in CD8^+^ T cells TRAIL can induce cell cycle arrest in G2/M phase (rather than apoptosis) [43]. In particular, this effect seems to depend on TRAIL-mediated cyclin B1 reduction, which would inhibit cyclin B1-dependent kinase and the transition from G2 to mitosis [44]. Nevertheless, TRAIL can reduce T cell proliferation also by inhibiting calcium influx, which leads to a downregulation of cyclin-dependent kinase 4 and to cell cycle progression blockade [45]. Not surprisingly, TRAIL is also involved in the regulation of plasma cell homeostasis, where it promotes apoptosis under specific conditions, which include the loss of both CD40 expression and NF*κ*B activation [46]. Consistent with this, administration of neutralizing anti-TRAIL antibodies markedly increases serum autoantibody levels in autoimmune prone CH3/HeJ gld/gld mice [47].

To complete the picture, TRAIL seems to have also anti-inflammatory actions. For example, Renshaw and colleagues demonstrated that neutrophil apoptosis is accelerated by leucine zipper-tagged TRAIL, which may represent a potential mechanism of neutrophil clearance at sites of inflammation [48]. Another argument in favour of TRAIL anti-inflammatory actions is what it does on atherosclerosis [49]. Atherosclerosis should in fact be considered as an inflammatory disease [50], since plaques are nothing but inflammatory aggregates, resulting from endothelial damage, which promotes subsequent smooth muscle cell migration and proliferation, monocyte-derived macrophage adhesion and accumulation to the site of injury, and fibrous tissue formation. In* in vitro* studies, TRAIL significantly reduced leukocyte/endothelial cell adhesion by downregulating CCL8 and CXCL10 chemokine expression [51]. In* in vivo* studies, TRAIL delivery reduced the extent of aortic atherosclerosis, possibly by inducing macrophage apoptosis [52], and consistent with this finding, Di Bartolo and colleagues reported a reduction in macrophage accumulation in the atherosclerotic plaques of TRAIL-knockout mice [53].

## 4. Protective Role of TRAIL against Diabetes

### 4.1. Type 1 Diabetes Mellitus and the Immune System

**Figure 2:**
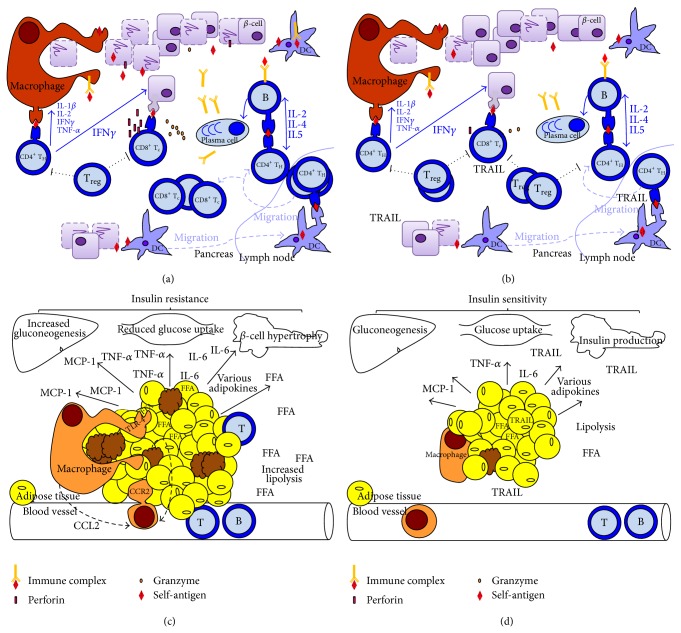
Current immunological view of T1DM and T2DM. (a) Islet inflammation in T1DM. A triggering insult recruits antigen-presenting cells (dendritic cells, macrophages, and B cells). Dendritic cells (DC) pick up self-antigens and immune complexes by pinocytosis and carry them to the pancreatic draining lymph nodes where they present them to autoreactive T cells and activate them. Such T cells migrate back to the islets where they destroy *β*-cells either by perforin, granzymes, or FasL-dependent interactions (CD8^+^), or by proinflammatory cytokine release (CD4^+^). Macrophages and B cells can also act as antigen-presenting cells. The cross-talk between B and T cells promotes the development of plasma cells, the release of autoantibodies, and the formation of immune complexes, which all together create a vicious cycle of inflammation and death. (b) TRAIL effects on islet inflammation. Experimental studies have shown that TRAIL inhibits T cell proliferation/expansion, induces T cell death, promotes Treg expansion, and protects *β*-cells. (c) Overview on obesity-induced insulin resistance. Obesity increases free fatty acids (FFA) that bind to toll-like receptor 4 (TLR-4) and activate adipose tissue macrophages. At the same time, the expansion of the adipose tissue, which outgrows its vascular supply, induces adipocyte necrosis, which stimulates macrophage recruitment and activation. One recruiting factor is CCL2, which mediates the recruitment of CCR2 monocytes, which differentiate into macrophages. Adipocyte death and innate immune cell activation stimulate the migration of adaptive immune cells. In addition, macrophage migration to the adipose tissue and their activation induce the expression of proinflammatory molecules, such as IL-6, MCP-1, TNF-*α*, and other adipokines which lead to insulin resistance. Peripheral insulin resistance is featured by impaired glucose uptake, increase of gluconeogenesis, hyperlipidemia, hyperglycemia, *β*-cell hypertrophy, *β*-cell inflammation, stress, and death. (d) TRAIL effects on obesity-induced insulin resistance. Experimental studies show that TRAIL reduces fat mass gain, systemic and tissue proinflammatory cytokines, and ameliorates peripheral insulin resistance. This is associated to a reduction of *β*-cell hypertrophy, inflammation, and loss.

Diabetes mellitus (DM) refers to a condition of hyperglycemia that can be further classified into T1DM, primarily due to a lack of insulin, or T2DM, primarily due to peripheral insulin resistance. Whether it is T1DM or T2DM, the immune system is now recognized to be crucially involved in the development and progression of both. T1DM is in fact a T cell-mediated autoimmune disease or according to Burnet and Mackay definition, “*a condition in which structural or functional damage is produced by the action of immunologically competent cells or antibodies against normal components of the body*” arising by “*the emergence of forbidden clones of T lymphocytes*” [54]. In particular, as depicted in Figure 2(a), genetic predisposition and unknown environmental factors lead to the release of *β*-cell DNA and antigens that promote the recruitment of macrophages, neutrophils, DC, and B cells, as well as the formation of autoantibodies in the pancreas [55]. These autoantibodies are usually directed against insulin, insulinoma-associated antigen (IA) 2, 65-kD isoform of glutamic acid decarboxylase (GAD-65), *β*-cell-specific zinc transporter (Znt8), and islet cell (ICA) 512, which are not only the major autoantibody targets but also the main epitopes activating autoreactive T cells [56]. Here DC pick up antigens and immune complexes by pinocytosis and, upon activation, migrate to the pancreatic lymph nodes to present *β*-cell antigens to an abnormal number of autoreactive T cells, which are there because of a defective immune tolerance. Then, autoreactive T cells become fully activated and home into the islets. It has to be noted that usually T cells can be activated by the recognition of autoantigens through MHC class II on antigen-presenting cells and/or through MHC class I on *β*-cells [57, 58]. Then, one way or the other, activated CD8^+^ T cells will destroy the *β*-cells by the release of cytolytic granules containing perforins and granzymes or by FasL-dependent interactions, while CD4^+^ release proinflammatory cytokines (Figure 2(a)). At the same time, inflammation and tissue damage are further worsened by an impairment of Treg cells, which seem to be either lacking [59] and/or defective [60] in T1DM. As for the natural history of the disease, this islet inflammation (or insulitis) generally precedes T1DM onset [61] and it corresponds to the stage where autoantibodies are detectable [62]. Of note, neither autoantibodies nor B cells seem to be critically involved in T1DM pathogenesis, as T1DM can affect patients with X-linked agammaglobulinemia [63]. Anyway, when 70–80% of *β*-cells have been destroyed, the residual insulin-producing cells are insufficient to maintain glucose tolerance and T1DM develops [64].

### 4.2. TRAIL and T1DM

Interestingly, experimental evidence suggests that TRAIL might protect against T1DM [65]. In the first animal study exploring this issue, TRAIL blockade by sDR5 injection significantly increased T1DM incidence and accelerated T1DM onset in NOD (nonobese diabetic) mice [66]. Moreover, in this study sDR5 injection led to a greater islet inflammation and it increased T cell proliferation as well as anti-GAD-65 levels [66]. Likewise, a second experiment showed that TRAIL deficiency worsened streptozotocin prodiabetogenic effects, since T1DM incidence and progression increased significantly in TRAIL-knockout mice [66]. Then, in the third work of the series, we showed that TRAIL delivery in streptozotocin-injected rats preserved pancreatic islets and significantly ameliorated the severity of T1DM, by lowering glucose levels [67]. These experimental findings are in line with the clinical observation that TRAIL circulating levels not only are decreased in T1DM patients but also are negatively correlated with patient insulin requirement, and the lowest levels of TRAIL are observed in those patients with diabetic ketoacidosis at onset [68].

Nevertheless, the mechanisms whereby TRAIL could defend from T1DM development and progression remain to be fully clarified. One hypothesis accounting for TRAIL protection against T1DM is that it could induce T cell death or inhibit their activation (Figure 2(b)). This has been partly confirmed by Mi and colleagues who showed that TRAIL suppressed the proliferation of autoreactive T cells isolated from diabetic NOD mice [69]. Here TRAIL upregulated p27 expression, which stops T cell cycle progression, and simultaneously inhibited IL-2, which on the contrary would promote p27 degradation and cell cycle progression [70]. Another hypothesis is that TRAIL could promote *β*-cell survival.* In vitro* experiments have confirmed that exposure to TRAIL does not affect *β*-cell viability in rat insulinoma cells. Here, on the contrary, TRAIL upregulates DcR1, which should prevent apoptosis [71] and has been interpreted as a defensive strategy of *β*-cells against infiltrating leukocytes [72]. In addition to that, TRAIL can induce the elevation of tissue inhibitor of metalloproteinase-1 (TIMP-1) [73], whose increase might protect against T1DM by reducing MMP-9 pancreatic activity [73], which cleaves insulin and is generally higher in diabetic patients [74–76]. This is consistent not only with* in vitro* experiments showing that the addition of TIMP-1 significantly reduces insulinoma cell death [73], but also with the finding that TIMP-1 prevents cytokine-mediated dysfunction and cytotoxicity in pancreatic islets and *β*-cells [77].

### 4.3. Type 2 Diabetes Mellitus and the Immune System

If T1DM is primarily an adaptive immune system-mediated autoimmune disease affecting the endocrine pancreas, T2DM and its complications seem to result – at least initially- from an activation of the innate immune system in the organs involved in glucose metabolism [78]. The theory that inflammation is involved in T2DM is in fact not new but dates back as far as to the end of the ninetieth century, when Ebstein reported that high doses of salicylate improved glycosuria in diabetic patients [79]. This theory was then abandoned until Pickup and Crook observed that the dyslipidemia found in T2DM patients (low HDL cholesterol and high triglycerides) is typically seen also in patients with acute-phase reactions and they put forward that lifestyle and the environment may cause T2DM in individuals with hypersensitive acute-phase response [80]. In line with these early observations, the current view on the human immunology of T2DM [81] is that inflammation links obesity to T2DM [81], as it mediates the development of obesity-induced insulin resistance in insulin target organs [82]. The first direct evidence of the connection existing between obesity, inflammation, and T2DM has been the elevation of TNF-*α*, a well-known proinflammatory molecule, in the plasma and adipose tissue of obese rodents, whose levels were proportional to insulin resistance, whose blockade ameliorated insulin sensitivity [83] and whose delivery recapitulated insulin resistance [84]. Consistent with it, also C-reactive protein, which is an unspecific measure of immune system activation, not only is associated with obesity [85] but also predicts [86] and/or identifies the people at risk of developing T2DM [87]. In line with these findings, we have recently shown that in high-fat diet fed mice, which become obese, there is not only an elevation of proinflammatory cytokines, but also of the TRAIL decoy receptor OPG, which promotes inflammatory changes and glucose abnormalities once it is delivered in control mice [88].

The explanation as to why obesity is associated with a prominent inflammatory response relies on the changes occurring in the white adipose tissue, which should be considered as an endocrine organ that changes its secretory pattern when it enlarges [89]. In particular, in obesity, fat mass expansion causes adipocyte hypoxia and necrosis because the adipose tissue outgrows its vascular supply. These events promote the secretion of proinflammatory molecules [90, 91], which all together stimulate macrophage recruitment and activation [82, 90, 92]. In addition to that, it has also been shown that in the fat of obese subjects there is an increased activity of 11*β*-hydroxysteroid dehydrogenase type 1 [93, 94], whose overexpression leads to proinflammatory changes in the adipocyte expression profile and to an increase in the number of macrophages [92]. It is macrophage infiltration into the expanding adipose tissue that is considered one of the key steps towards insulin resistance and T2DM development [95]. Once macrophages home to the inflamed fat mass, circulating and tissue proinflammatory cytokines increase, which will inhibit insulin signalling in peripheral tissues, leading to obesity-related insulin resistance (Figure 2(c)) [96]. Consistent with it, high-fat diet fed mice lacking the C-C motif chemokine receptor-2 that is essential for macrophage recruitment exhibited fewer macrophages and a lower inflammatory gene profile in the adipose tissue as well as reduced insulin resistance [97]. Conversely, mice overexpressing MCP-1 in their adipose tissue showed the opposite phenotype [98]. At this stage, the inflamed fat mass stimulates the migration of T and B cells, both contributing to insulin resistance [99]. Obesity-related insulin resistance, however, affects not only adipose tissue but also all insulin target organs. In the fat lipolysis and free fatty acid (FFA) levels increase, stimulating tissue inflammation and damage, for example, by activating toll-like receptor 4 (TLR-4) [100], while in the liver gluconeogenesis increases and in skeletal muscle glucose uptake is reduced. Hyperlipidemia and hyperglycemia stimulate *β*-cell hypertrophy and hyperinsulinemia, ultimately leading to *β*-cell stress, exhaustion, inflammation, and T2DM.

### 4.4. TRAIL and T2DM

**Figure 3:**
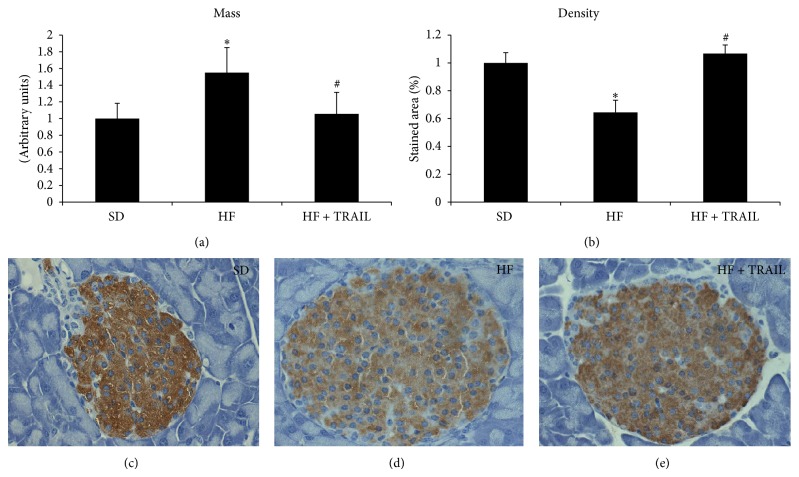
TRAIL reduces *β*-cell mass and increases *β*-cell density in the islets of HFD fed mice. Quantification of mass and density and representative histological sections of pancreas islets from standard diet (SD), high-fat diet (HF), or high-fat diet fed mice treated with TRAIL (HF + TRAIL) (magnification 40x). The sections were stained with polyclonal antibody against insulin (3,3′-diaminobenzidine-DAB) and counterstained with hematoxylin. Pancreatic *β*-cell mass was estimated by multiplying the mean density of staining for insulin in the islet section by the mean islet area per area of pancreas. This was expressed in arbitrary units adjusted for the wet weight of the pancreas of each mouse. Pancreatic *β*-cell density was estimated by determining the percentage proportion of islet area occupied by the brown (DAB) staining and was expressed as percentage stained area. Data are mean ± SEM ^*^ *P* < 0.05 versus SD; ^#^ *P* < 0.05 versus HF.

**Figure 4:**
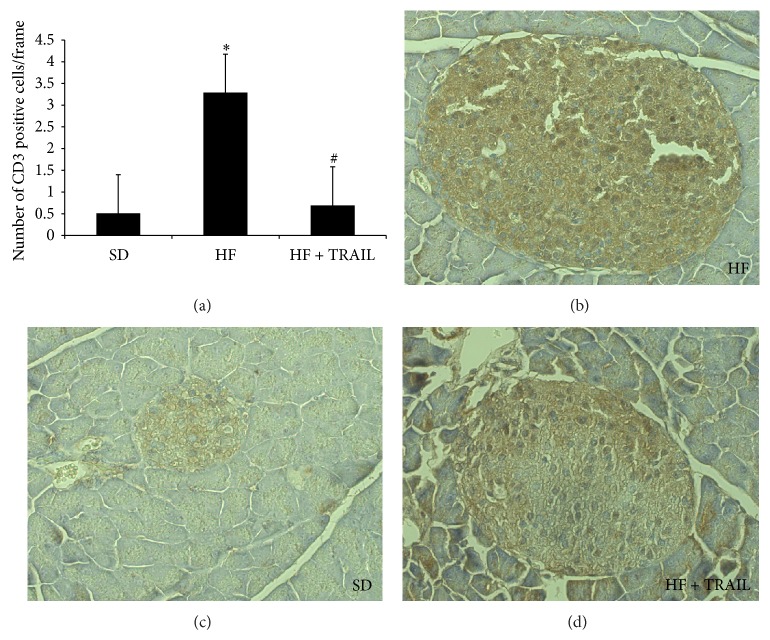
TRAIL reduces CD3 infiltration. CD3 count and representative histological sections of pancreas islets from standard diet (SD), high-fat diet (HF), or high-fat diet fed mice treated with TRAIL (HF + TRAIL) (magnification 20x). The sections were stained with polyclonal antibody against CD3 (3,3′-diaminobenzidine-DAB) and counterstained with hematoxylin. Pancreatic CD3 infiltration was estimated by determining the total number of cells positive for CD3 per islet. Data are mean ± SEM ^*^ *P* < 0.01 versus SD; ^#^ *P* < 0.01 versus HF.

Animal studies suggest that TRAIL might protect against T2DM too. Di Bartolo and colleagues found that TRAIL deficiency promoted diabetes development in ApoE/TRAIL-knockout mice put on a high-fat diet [53], which is commonly used to induce obesity and insulin resistance in rodents [88, 101, 102]. In particular, ApoE/TRAIL-knockout mice displayed significantly higher glucose levels and lower insulin levels than ApoE-knockout mice. Consequently, this study showed that TRAIL deficiency significantly worsened glucose tolerance, and this was associated with *β*-cell function impairment, *β*-cell density reduction, and an increase in islet macrophage infiltration. Consistent with this we have documented the ability of TRAIL to significantly reverse the metabolic abnormalities due to an oversupply of lipids and thus to slow down the natural progression of T2DM [101]. In particular, we studied 27 male C57Bl6J mice randomly allocated to standard diet (SD), high-fat diet (HF), and high-fat diet + TRAIL (HF + TRAIL) for 12 weeks. TRAIL was delivered weekly by intraperitoneal injection at a dose of 10 *μ*g per mouse. At the end of the study we found that TRAIL treatment reduced significantly the hyperglycemia and the hyperinsulinemia displayed by the high-fat diet fed mice during an IPGTT. Moreover, TRAIL treatment significantly reduced the *β*-cell hypertrophy and the *β*-cell loss, as it reduced *β*-cell mass and increased *β*-cell density (Figure 3). This was associated with a decrease in protein nitrosylation (data not shown), as well as with a significant reduction in CD3 infiltration (Figure 4) in the islets of TRAIL treated high-fat diet fed mice. This is of relevance since lymphocyte-driven autoimmune assault is one of the mechanisms accounting for *β*-cell destruction in T2DM [96]. Interestingly, we have also shown that OPG, which is TRAIL decoy receptor, has opposite actions to those of TRAIL, as it seems to promote glucose intolerance [88, 103].

The mechanisms underlying TRAIL protection against T2DM development may include some antiadipogenic and anti-inflammatory effects of this molecule (Figure 2(d)). Going back to the adipose tissue, the demonstration that its removal improves insulin resistance highlights the crucial role that the fat plays in the development of obesity-induced insulin resistance and T2DM [104]. So, the first mechanism whereby TRAIL could protect against T2DM is its effect on adiposity. In our study TRAIL significantly reduced the deposition of fat mass induced by the high-fat diet [101], due to proapoptotic and antiadipogenic actions, as it induced the expression of caspase-3 while it inhibited that of PPAR-*γ*. This is consistent with what has been reported by Di Bartolo and colleagues, who found that mice lacking TRAIL presented an increased deposition of fat [53]. Moreover, human studies show that there is a correlation between circulating TRAIL and body mass [105], which may be interpreted as a compensatory mechanism set up by the body to respond to fat gain and which in any case reinforces the notion that TRAIL is linked to body weight regulation. In the second place, TRAIL has also the ability to put out inflammation, which may be the consequence of its antiadipogenic actions, or of its effects on the immune system. In particular, we found that TRAIL significantly reduced systemic and tissue IL-6, MCP-1, and TNF-*α* [101], which on the contrary were elevated in TRAIL deficient mice [53]. This could explain the reduction in islet CD3 cells that we observed in TRAIL treated mice and the amelioration of their metabolic abnormalities.

## 5. Conclusion

There is abundant literature showing that TRAIL is involved not only in tumor growth suppression and/or infection control, but also in the regulation of both innate and adaptive immune system. Recently, a few* in vivo* studies have demonstrated that TRAIL might protect against the development and/or progression of diabetes. Therefore, since the immune system is crucially involved in diabetes development, it is through its action on innate and adaptive immunity that TRAIL is likely to protect against diabetes. TRAIL is in fact involved in the regulation of central and peripheral tolerance, mediates T cell death and inhibits their proliferation, and promotes Treg cells. As for T2DM, TRAIL seems to prevent fat mass enlargement and exerts anti-inflammatory actions, whereby it could ameliorate the metabolic abnormalities typical of obesity-induced insulin resistance. The idea that this molecule could kill what is leading/contributing to diabetes, such as activated immune cells, macrophages, or even adipocytes, and protect normal *β*-cells is truly fascinating. Nevertheless further* in vivo* studies are needed to clarify the extent of TRAIL actions and further* in vitro* studies in order to clarify its precise mechanisms of action.

## References

[1] Bernardi S., Secchiero P., Zauli G. (2012). State of art and recent developments of anti-cancer strategies based on TRAIL. *Recent Patents on Anti-Cancer Drug Discovery*.

[2] Wiley S. R., Schooley K., Smolak P. J. (1995). Identification and characterization of a new member of the TNF family that induces apoptosis. *Immunity*.

[3] Nikoletopoulou V., Markaki M., Palikaras K., Tavernarakis N. (2013). Crosstalk between apoptosis, necrosis and autophagy. *Biochimica et Biophysica Acta*.

[4] Jouan-Lanhouet S., Arshad M. I., Piquet-Pellorce C. (2012). TRAIL induces necroptosis involving RIPK1/RIPK3-dependent PARP-1 activation. *Cell Death and Differentiation*.

[5] Pan G., O'Rourke K., Chinnaiyan A. M. (1997). The receptor for the cytotoxic ligand TRAIL. *Science*.

[6] Pan G., Ni J., Wei Y.-F., Yu G.-I., Gentz R., Dixit V. M. (1997). An antagonist decoy receptor and a death domain-containing receptor for TRAIL. *Science*.

[7] Sheridan J. P., Marsters S. A., Pitti R. M. (1997). Control of TRAIL-induced apoptosis by a family of signaling and decoy receptors. *Science*.

[8] Wu G. S., Burns T. F., McDonald E. R. (1997). KILLER/DR5 is a DNA damage-inducible p53-regulated death receptor gene. *Nature Genetics*.

[9] Milani D., Zauli G., Rimondi E. (2003). Tumour necrosis factor-related apoptosis-inducing ligand sequentially activates pro-survival and pro-apoptotic pathways in SK-N-MC neuronal cells. *Journal of Neurochemistry*.

[10] Degli-Esposti M. A., Smolak P. J., Walczak H. (1997). Cloning and characterization of TRAIL-R3, a novel member of the emerging TRAIL receptor family. *The Journal of Experimental Medicine*.

[11] Degli-Esposti M. A., Dougall W. C., Smolak P. J., Waugh J. Y., Smith C. A., Goodwin R. G. (1997). The novel receptor TRAIL-R4 induces NF-*κ*B and protects against TRAIL- mediated apoptosis, yet retains an incomplete death domain. *Immunity*.

[12] Marsters S. A., Sheridan J. P., Pitti R. M. (1997). A novel receptor for Apo2L/TRAIL contains a truncated death domain. *Current Biology*.

[13] Zauli G., Melloni E., Capitani S., Secchiero P. (2009). Role of full-length osteoprotegerin in tumor cell biology. *Cellular and Molecular Life Sciences*.

[14] Cretney E., Takeda K., Yagita H., Glaccum M., Peschon J. J., Smyth M. J. (2002). Increased susceptibility to tumor initiation and metastasis in TNF-related apoptosis-inducing ligand-deficient mice. *Journal of Immunology*.

[15] Sedger L. M., Glaccum M. B., Schuh J. C. (2002). Characterization of the in vivo function of TNF-*α*-related apoptosis-inducing ligand, TRAIL/Apo2L, using TRAIL/Apo2L gene-deficient mice. *European Journal of Immunology*.

[16] Janssen E. M., Droin N. M., Lemmens E. E. (2005). CD4^+^ T-cell help controls CD8^+^ T-cell memory via TRAIL-mediated activation-induced cell death. *Nature*.

[17] Takeda K., Smyth M. J., Cretney E. (2002). Critical role for tumor necrosis factor-related apoptosis-inducing ligand in immune surveillance against tumor development. *The Journal of Experimental Medicine*.

[18] Huntington N. D., Vosshenrich C. A. J., di Santo J. P. (2007). Developmental pathways that generate natural-killer-cell diversity in mice and humans. *Nature Reviews Immunology*.

[19] Nagata S. (1997). Apoptosis by death factor. *Cell*.

[20] Tartaglia L. A., Goeddel D. V. (1992). Two TNF receptors. *Immunology Today*.

[21] Ashkenazi A., Herbst R. S. (2008). To kill a tumor cell: the potential of proapoptotic receptor agonists. *The Journal of Clinical Investigation*.

[22] Ashkenazi A., Pai R. C., Fong S. (1999). Safety and antitumor activity of recombinant soluble Apo2 ligand. *The Journal of Clinical Investigation*.

[23] Leverkus M., Walczak H., McLellan A. (2000). Maturation of dendritic cells leads to up-regulation of cellular FLICE-inhibitory protein and concomitant down-regulation of death ligand-mediated apoptosis. *Blood*.

[24] Secchiero P., Gonelli A., Carnevale E. (2003). TRAIL promotes the survival and proliferation of primary human vascular endothelial cells by activating the Akt and ERK pathways. *Circulation*.

[25] Zauli G., Sancilio S., Cataldi A., Sabatini N., Bosco D., Di Pietro R. (2005). PI-3K/Akt and NF-*κ*B/I*κ*B*α* pathways are activated in Jurkat T cells in response to TRAIL treatment. *Journal of Cellular Physiology*.

[26] Hunter I., Nixon G. F. (2006). Spatial compartmentalization of tumor necrosis factor (TNF) receptor 1-dependent signaling pathways in human airway smooth muscle cells: lipid rafts are essential for TNF-*α*-mediated activation of RhoA but dispensable for the activation of the NF-*κ*B and MAPK pathways. *The Journal of Biological Chemistry*.

[27] Song J. H., Tse M. C. L., Bellail A. (2007). Lipid rafts and nonrafts mediate tumor necrosis factor-related apoptosis-inducing ligand-induced apoptotic and nonapoptotic signals in non-small cell lung carcinoma cells. *Cancer Research*.

[28] Toffoli B., Bernardi S., Candido R., Zacchigna S., Fabris B., Secchiero P. (2012). TRAIL shows potential cardioprotective activity. *Investigational New Drugs*.

[29] Sedger L. M., Katewa A., Pettersen A. K. (2010). Extreme lymphoproliferative disease and fatal autoimmune thrombocytopenia in FasL and TRAIL double-deficient mice. *Blood*.

[30] Lamhamedi-Cherradi S.-E., Zheng S.-J., Maguschak K. A., Peschon J., Chen Y. H. (2003). Defective thymocyte apoptosis and accelerated autoimmune diseases in TRAIL^−/−^ mice. *Nature Immunology*.

[31] Cretney E., Uldrich A. P., Berzins S. P., Strasser A., Godfrey D. I., Smyth M. J. (2003). Normal thymocyte negative selection in TRAIL-deficient mice. *The Journal of Experimental Medicine*.

[32] Walsh C. M., Wen B. G., Chinnaiyan A. M., O'Rourke K., Dixit V. M., Hedrick S. M. (1998). A role for FADD in T cell activation and development. *Immunity*.

[33] Corazza N., Brumatti G., Jakob S., Villunger A., Brunner T. (2004). TRAIL and thymocyte apoptosis: not so deadly?. *Cell Death and Differentiation*.

[34] Marsters S. A., Pitti R. M., Donahue C. J., Ruppert S., Bauer K. D., Ashkenazi A. (1996). Activation of apoptosis by Apo-2 ligand is independent of FADD but blocked by CrmA. *Current Biology*.

[35] Martínez-Lorenzo M. J., Alava M. A., Gamen S. (1998). Involvement of APO2 ligand/TRAIL in activation-induced death of Jurkat and human peripheral blood T cells. *European Journal of Immunology*.

[36] Ikeda T., Hirata S., Fukushima S. (2010). Dual effects of TRAIL in suppression of autoimmunity: the inhibition of Th1 cells and the promotion of regulatory T cells. *Journal of Immunology*.

[37] Wang S. H., Chen G. H., Fan Y., Van Antwerp M., Baker J. R. (2009). Tumor necrosis factor-related apoptosis-inducing ligand inhibits experimental autoimmune thyroiditis by the expansion of CD4^+^CD25^+^ regulatory T cells. *Endocrinology*.

[38] Sakaguchi S., Powrie F., Ransohoff R. M. (2012). Re-establishing immunological self-tolerance in autoimmune disease. *Nature Medicine*.

[39] Lehnert C., Weiswange M., Jeremias I. (2014). TRAIL-receptor costimulation inhibits proximal TCR signaling and suppresses human T cell activation and proliferation. *The Journal of Immunology*.

[40] Song K., Chen Y., Göke R. (2000). Tumor necrosis factor-related apoptosis-inducing ligand (TRAIL) is an inhibitor of autoimmune inflammation and cell cycle progression. *Journal of Experimental Medicine*.

[41] Zhang X. R., Zhang L. Y., Devadas S., Li L., Keegan A. D., Shi Y. F. (2003). Reciprocal expression of TRAIL and CD95L in Th1 and Th2 cells: role of apoptosis in T helper subset differentiation. *Cell Death & Differentiation*.

[42] Roberts A. I., Devadas S., Zhang X. (2003). The role of activation-induced cell death in the differentiation of T-helper-cell subsets. *Immunologic Research*.

[43] Bosque A., Pardo J., Martínez-Lorenzo M. J. (2005). Human CD8^+^ T cell blasts are more sensitive than CD4^+^ T cell blasts to regulation by APO2L/TRAIL. *European Journal of Immunology*.

[44] Bosque A., Aguiló J. I., Del Rey M. (2008). Cell cycle regulation by FasL and Apo2L/TRAIL in human T-cell blasts. Implications for autoimmune lymphoproliferative syndromes. *Journal of Leukocyte Biology*.

[45] Lünemann J. D., Waiczies S., Ehrlich S. (2002). Death ligand TRAIL induces no apoptosis but inhibits activation of human (auto)antigen-specific T cells. *Journal of Immunology*.

[46] Ursini-Siegel J., Zhang W., Altmeyer A. (2002). Trail/Apo-2 ligand induces primary plasma cell apoptosis. *The Journal of Immunology*.

[47] Kayagaki N., Yamaguchi N., Abe M. (2002). Suppression of antibody production by TNF-related apoptosis-inducing ligand (TRAIL). *Cellular Immunology*.

[48] Renshaw S. A., Parmar J. S., Singleton V. (2003). Acceleration of human neutrophil apoptosis by TRAIL. *Journal of Immunology*.

[49] Bernardi S., Milani D., Fabris B., Secchiero P., Zauli G. (2012). TRAIL as biomarker and potential therapeutic tool for cardiovascular diseases. *Current Drug Targets*.

[50] Ross R. (1999). Atherosclerosis—an inflammatory disease. *The New England Journal of Medicine*.

[51] Secchiero P., Corallini F., Di Iasio M. G., Gonelli A., Barbarotto E., Zauli G. (2005). TRAIL counteracts the proadhesive activity of inflammatory cytokines in endothelial cells by down-modulating CCL8 and CXCL10 chemokine expression and release. *Blood*.

[52] Secchiero P., Candido R., Corallini F. (2006). Systemic tumor necrosis factor-related apoptosis-inducing ligand delivery shows antiatherosclerotic activity in apolipoprotein E-null diabetic mice. *Circulation*.

[53] Di Bartolo B. A., Chan J., Bennett M. R. (2011). TNF-related apoptosis-inducing ligand (TRAIL) protects against diabetes and atherosclerosis in Apoe ^−/−^ mice. *Diabetologia*.

[54] Roberts-Thomson P. J., Jackson M. W., Gordon T. P. (2012). A seminal monograph: Mackay and Burnet's Autoimmune diseases. *Medical Journal of Australia*.

[55] Diana J., Simoni Y., Furio L. (2013). Crosstalk between neutrophils, B-1a cells and plasmacytoid dendritic cells initiates autoimmune diabetes. *Nature Medicine*.

[56] Koczwara K., Bonifacio E., Ziegler A.-G. (2004). Transmission of maternal islet antibodies and risk of autoimmune diabetes in offspring of mothers with type 1 diabetes. *Diabetes*.

[57] Di Lorenzo T. P., Peakman M., Roep B. O. (2007). Translational mini-review series on type 1 diabetes: systematic analysis of T cell epitopes in autoimmune diabetes. *Clinical & Experimental Immunology*.

[58] Hamilton-Williams E. E., Palmer S. E., Charlton B., Slattery R. M. (2003). Beta cell MHC class I is a late requirement for diabetes. *Proceedings of the National Academy of Sciences of the United States of America*.

[59] Buschard K., Madsbad S., Rygaard J. (1980). Depressed suppressor cell activity in patients with newly diagnosed insulin-dependent diabetes mellitus. *Clinical and Experimental Immunology*.

[60] Roncarolo M.-G., Battaglia M. (2007). Regulatory T-cell immunotherapy for tolerance to self antigens and alloantigens in humans. *Nature Reviews Immunology*.

[61] Roep B. O. (2003). The role of T-cells in the pathogenesis of Type 1 diabetes: from cause to cure. *Diabetologia*.

[62] Eisenbarth G. S. (1986). Type I diabetes mellitus. A chronic autoimmune disease. *The New England Journal of Medicine*.

[63] Martin S., Wolf-Eichbaum D., Duinkerken G. (2001). Development of type 1 diabetes despite severe hereditary B-cell deficiency. *The New England Journal of Medicine*.

[64] van Belle T. L., Coppieters K. T., von Herrath M. G. (2011). Type 1 diabetes: etiology, immunology, and therapeutic strategies. *Physiological Reviews*.

[65] Bernardi S., Norcio A., Toffoli B., Zauli G., Secchiero P. (2012). Potential role of TRAIL in the management of autoimmune diabetes mellitus. *Current Pharmaceutical Design*.

[66] Lamhamedi-Cherradi S. E., Zheng S., Tisch R. M., Chen Y. H. (2003). Critical roles of tumor necrosis factor-related apoptosis-inducing ligand in type 1 diabetes. *Diabetes*.

[67] Zauli G., Toffoli B., Di Iasio M. G., Celeghini C., Fabris B., Secchiero P. (2010). Treatment with recombinant tumor necrosis factor-related apoptosis-inducing ligand alleviates the severity of streptozotocin-induced diabetes. *Diabetes*.

[68] Tornese G., Iafusco D., Monasta L. (2014). The levels of circulating TRAIL at the onset of type 1 diabetes are markedly decreased in patients with ketoacidosis and with the highest insulin requirement. *Acta Diabetologica*.

[69] Mi Q.-S., Ly D., Lamhamedi-Cherradi S.-E. (2003). Blockade of tumor necrosis factor-related apoptosis-inducing ligand exacerbates type 1 diabetes in NOD mice. *Diabetes*.

[70] Appleman L. J., Tzachanis D., Grader-Beck T., van Puijenbroek A. A. F. L., Boussiotis V. A. (2000). Helper T cell anergy: from biochemistry to cancer pathophysiology and therapeutics. *Journal of Molecular Medicine*.

[71] Kang S., Park S.-Y., Lee H.-J., Yoo Y. H. (2010). TRAIL upregulates decoy receptor 1 and mediates resistance to apoptosis in insulin-secreting INS-1 cells. *Biochemical and Biophysical Research Communications*.

[72] Dirice E., Kahraman S., Elpek G. O. (2011). TRAIL and DcR1 expressions are differentially regulated in the pancreatic islets of STZ- versus CY-applied NOD mice. *Experimental Diabetes Research*.

[73] Kang S., Park E.-J., Joe Y. (2010). Systemic delivery of TNF-Related Apoptosis-Inducing Ligand (TRAIL) elevates levels of tissue inhibitor of metalloproteinase-1 (TIMP-1) and prevents type 1 diabetes in nonobese diabetic mice. *Endocrinology*.

[74] Maxwell P. R., Timms P. M., Chandran S., Gordon D. (2001). Peripheral blood level alterations of TIMP-1, MMP-2 and MMP-9 in patients with Type 1 diabetes. *Diabetic Medicine*.

[75] Descamps F. J., Martens E., Ballaux F., Geboes K., Opdenakker G. (2004). In vivo activation of gelatinase B/MMP-9 by trypsin in acute pancreatitis is a permissive factor in streptozotocin-induced diabetes. *Journal of Pathology*.

[76] Xue M., Thompson P. J., Clifton-Bligh R., Fulcher G., Gallery E. D. M., Jackson C. (2005). Leukocyte matrix metalloproteinase-9 is elevated and contributes to lymphocyte activation in type I diabetes. *International Journal of Biochemistry and Cell Biology*.

[77] Han X., Sun Y., Scott S., Bleich D. (2001). Tissue inhibitor of metalloproteinase-1 prevents cytokine-mediated dysfunction and cytotoxicity in pancreatic islets and *β*-cells. *Diabetes*.

[78] Defronzo R. A. (2009). Banting lecture. From the triumvirate to the ominous octet: a new paradigm for the treatment of type 2 diabetes mellitus. *Diabetes*.

[79] Ebstein W. (1876). Zur therapie des diabetes mellitus, insbesondere uber die Anwendung des salicylsauren natron bei demselben. *Berliner Klinische Wochenschrift*.

[80] Pickup J. C., Crook M. A. (1998). Is type II diabetes mellitus a disease of the innate immune system?. *Diabetologia*.

[81] Yang W., Lu J., Weng J. (2010). Prevalence of diabetes among men and women in China. *The New England Journal of Medicine*.

[82] Tataranni P. A., Ortega E. (2005). A burning question: does an adipokine-induced activation of the immune system mediate the effect of overnutrition on type 2 diabetes?. *Diabetes*.

[83] Hotamisligil G. S., Shargill N. S., Spiegelman B. M. (1993). Adipose expression of tumor necrosis factor-*α*: direct role in obesity-linked insulin resistance. *Science*.

[84] Hotamisligil G. S., Peraldi P., Budavari A., Ellis R., White M. F., Spiegelman B. M. (1996). IRS-1-mediated inhibition of insulin receptor tyrosine kinase activity in TNF-*α*- and obesity-induced insulin resistance. *Science*.

[85] Visser M., Bouter L. M., McQuillan G. M., Wener M. H., Harris T. B. (1999). Elevated C-reactive protein levels in overweight and obese adults. *The Journal of the American Medical Association*.

[86] Freeman D. J., Norrie J., Caslake M. J. (2002). C-reactive protein is an independent predictor of risk for the development of diabetes in the west of Scotland coronary prevention study. *Diabetes*.

[87] Hu F. B., Meigs J. B., Li T. Y., Rifai N., Manson J. E. (2004). Inflammatory markers and risk of developing type 2 diabetes in women. *Diabetes*.

[88] Bernardi S., Fabris B., Thomas M. (2014). Osteoprotegerin increases in metabolic syndrome and promotes adipose tissue proinflammatory changes. *Molecular and Cellular Endocrinology*.

[89] Sell H., Habich C., Eckel J. (2012). Adaptive immunity in obesity and insulin resistance. *Nature Reviews Endocrinology*.

[90] Cinti S., Mitchell G., Barbatelli G. (2005). Adipocyte death defines macrophage localization and function in adipose tissue of obese mice and humans. *Journal of Lipid Research*.

[91] Strissel K. J., Stancheva Z., Miyoshi H. (2007). Adipocyte death, adipose tissue remodeling, and obesity complications. *Diabetes*.

[92] Weisberg S. P., McCann D., Desai M., Rosenbaum M., Leibel R. L., Ferrante A. W. (2003). Obesity is associated with macrophage accumulation in adipose tissue. *Journal of Clinical Investigation*.

[93] Lindsay R. S., Wake D. J., Nair S. (2003). Subcutaneous adipose 11*β*-hydroxysteroid dehydrogenase type 1 activity and messenger ribonucleic acid levels are associated with adiposity and insulinemia in Pima Indians and Caucasians. *The Journal of Clinical Endocrinology & Metabolism*.

[94] Masuzaki H., Paterson J., Shinyama H. (2001). A transgenic model of visceral obesity and the metabolic syndrome. *Science*.

[95] Xu H., Barnes G. T., Yang Q. (2003). Chronic inflammation in fat plays a crucial role in the development of obesity-related insulin resistance. *The Journal of Clinical Investigation*.

[96] Odegaard J. I., Chawla A. (2012). Connecting type 1 and type 2 diabetes through innate immunity. *Cold Spring Harbor Perspectives in Medicine*.

[97] Weisberg S. P., Hunter D., Huber R. (2006). CCR2 modulates inflammatory and metabolic effects of high-fat feeding. *Journal of Clinical Investigation*.

[98] Kanda H., Tateya S., Tamori Y. (2006). MCP-1 contributes to macrophage infiltration into adipose tissue, insulin resistance, and hepatic steatosis in obesity. *The Journal of Clinical Investigation*.

[99] Winer S., Chan Y., Paltser G. (2009). Normalization of obesity-associated insulin resistance through immunotherapy. *Nature Medicine*.

[100] Saberi M., Woods N.-B., de Luca C. (2009). Hematopoietic cell-specific deletion of toll-like receptor 4 ameliorates hepatic and adipose tissue insulin resistance in high-fat-fed mice. *Cell Metabolism*.

[101] Bernardi S., Zauli G., Tikellis C. (2012). TNF-related apoptosis-inducing ligand significantly attenuates metabolic abnormalities in high-fat-fed mice reducing adiposity and systemic inflammation. *Clinical Science (London, England : 1979)*.

[102] Bernardi S., Tikellis C., Candido R. (2015). ACE2 deficiency shifts energy metabolism towards glucose utilization. *Metabolism*.

[103] Toffoli B., Bernardi S., Candido R. (2011). Osteoprotegerin induces morphological and functional alterations in mouse pancreatic islets. *Molecular and Cellular Endocrinology*.

[104] Pitombo C., Araújo E. P., De Souza C. T., Pareja J. C., Geloneze B., Velloso L. A. (2006). Amelioration of diet-induced diabetes mellitus by removal of visceral fat. *Journal of Endocrinology*.

[105] Harith H. H., Morris M. J., Kavurma M. M. (2013). On the TRAIL of obesity and diabetes. *Trends in Endocrinology and Metabolism*.

